# Is Corticalization in Radiographs Related to a Higher Risk of Bone Loss around Dental Implants in Smoking Patients? A 5-Year Observation of Radiograph Bone-Texture Changes

**DOI:** 10.3390/jcm12165351

**Published:** 2023-08-17

**Authors:** Tomasz Wach, Piotr Hadrowicz, Grzegorz Trybek, Adam Michcik, Marcin Kozakiewicz

**Affiliations:** 1 Department of Maxillofacial Surgery, Medical University of Lodz, 113 Żeromskiego Str., 90-549 Lodz, Poland; marcin.kozakiewicz@umed.lodz.pl; 2 Department of Otolaryngology, Hospital in Sosnowiec, Zegadłowicza 3, 41-200 Sosnowiec, Poland; phadrowicz@gmail.com; 3 Department of Oral Surgery, Pomeranian Medical University in Szczecin, 70-111 Szczecin, Poland; g.trybek@gmail.com; 4 4th Military Clinical Hospital in Wroclaw, ul. Rudolfa Weigla 5, 50-981 Wroclaw, Poland; 5 Department of Maxillofacial Surgery, Medical University of Gdansk, 80-210 Gdańsk, Poland; adammichcik@gumed.edu.pl

**Keywords:** bone remodeling, dental implant, smoking, torque, marginal bone loss, intraoral radiographs, radiomics, texture analysis

## Abstract

Background: Currently, the topic of dental implants is widely researched. However, still compromising are the factors that can affect implant loss as a consequence of marginal bone loss. One of the factors is smoking, which has a devastating effect on human health and bone structure. Oral health and jaw condition are also negatively affected by smoking. The aim of this study was to evaluate the peri-implant jawbone corticalization phenomenon in tobacco smokers. Methods: A total of 2196 samples from 768 patients with an implant in the neck area were checked, and texture features were analyzed. The corticalization phenomenon was investigated. All analyses were performed in MaZda Software. The influence of corticalization was investigated as a factor on bone structure near the implant neck. The statistical analysis included a feature distribution evaluation, mean (*t*-test) or median (W-test) comparison, analysis of regression and one-way analysis of variance or Kruskal–Wallis test as no normal distribution or between-group variance was indicated for the significant differences in the investigated groups. Detected differences or relationships were assumed to be statistically significant when *p* < 0.05. Results: The research revealed that MBL was correlated with smoking after 5 years (0.42 mm ± 1.32 mm 0 mm ± 1.25 mm), the Corticalization Index was higher in the smoker group on the day of surgery, and it became higher after 5y of observation (185.98 ± 90.8 and 243.17 ± 155.47). The implant-loss frequency was higher in the group of smokers, too, compared to non-smokers (6.74% and 2.87%). The higher the torque value during the implant placement, the higher the Corticalization Phenomenon Index. Conclusions: The research revealed a correlation between smoking and changes in bone structure in radio textures near the implants. The corticalization phenomenon is important, may be detected immediately after implant placement and may be one of the indicators of the implant success rate.

## 1. Introduction

Tobacco smoking has a devastating effect on human health and is one of the leading risks of early death. Smoking was the second risk factor for deaths in the world in 2019. Nearly 7.69 million people died because of this addiction [1,2,3]. There are many health disorders caused by smoking. For example, it increases the risk of many types of cancer and heart and lung diseases and increases the risk of pregnancy complications, oral cavity diseases, neuroticism (also in groups of e-cigarette smokers) and more [4,5,6,7,8,9]. Marginal bone loss is strongly connected with smoking and also with the degree of smoking [10,11,12,13,14]. On the other hand, it is not stated that tobacco smoking is an absolute contraindication for implant placement, but it is still associated with decreased dental implant surveys [14,15].

Bone metabolism is also disturbed directly and indirectly. Smoking directly decreases osteogenesis and angiogenesis and, through this, increases osteoclast cell activity and bone resorption occur. Indirectly, it causes decreased parathormone levels, estrogen production and levels of oxidants. It increases the level of cortisol, as well as free radicals. All of the above have an indirect negative effect on the balance of osteoblasts/osteoclasts activity [16,17,18], such as bisphosphonates’ action [19,20,21]. Spine densitometry is one of the methods used to check and control the metabolism of human bone [22,23]. Previous studies observed a warning relation between marginal bone loss and the transformation of the bone surrounding dental implants toward corticalization [24,25]. The coexistence of these two phenomena may have an adverse effect on the long-term success of dental implant treatment.

This raised the question of whether a known negative factor (tobacco smoking) increases corticalization.

The aim of this study was to evaluate peri-implant jawbone corticalization in tobacco smokers.

## 2. Materials and Methods

This research is the result of analyses of prospective radiological data of oral implantological treatment of patients with dental loss.

The inclusion criteria were as follows: at least 18 years of age, bleeding upon gingival probing < 20%, probing depth ≤ 3 mm, good oral hygiene, regular follow-ups, two-dimensional radiographs taken during regular checks, laboratory tests to check vitamin levels and ions and hormones levels—parathormone (PTH, where the norm is 10 to 60 pg/mL); thyrotropin (TSH), where the norm is 0.23–4.0 µU/mL; calcium in serum (Ca^2+^), where the norm is 9–11 mg/dL; glycated hemoglobin (HbA1c), where the norm is <5%; and vitamin 25(OH)D3 (D3), where the norm is 31–50 ng/mL. Patients smoking 1 or more cigarettes per day were qualified to be in the smoker group.

Spine densitometry, where the T-score can be examined, was also considered. The T-score shows the ratio between bone mineral density (BMD) of the examined patient and the average BMD for young patients. The normal value for normal bone is >−1.0, osteopenia is indicated by values between −1.0 and −2.5, and scores <−2.5 indicate osteoporosis (Table 1).

The exclusion criteria included a lack of or defective X-ray images in the visual assessment, lack of laboratory tests, uncontrolled internal co-morbidity (diabetes mellitus, thyroid dishormonoses, rheumatoid disease and other immunodeficiencies), a history of oral radiation therapy, past or current use of cytostatic drugs, soft and bone tissue augmentation, and low-quality or lack of follow-up radiographs. Finally, 2196 samples of a dental implant neck area (768 patients) were included in this study and analyzed (the average number of implants per patient was 2.86) (Table 2).

Additionally, the following factors were considered: confidence level of 95%, margin error of 5%, population of 37 million and a fraction of 28.8%. In that case, the minimal sample size is 323 patients. Taking into account the amount of 768 patients, the error is on the level of 3%.

The limitation of the study is that the laboratory tests have not been carried out 3 months after the research (Table 3).

Twenty-two types of dental implants were used in this study: AB Dental Devices I5 (www.ab-dent.com Ashdod, Israel; accessed on 21 July 2022); ADIN Dental Implants Touareg (www.adin-implants.com Afula, Israel; accessed on 21 July 2022); Alpha Bio ARRP (www.alpha-bio.net Petah-Tikva, Israel; accessed on 21 July 2022); Alpha Bio ATI (www.alpha-bio.net Petah-Tikva, Israel; accessed on 21 July 2022); Alpha Bio DFI (www.alpha-bio.net Petah-Tikva, Israel); Alpha Bio OCI (www.alpha-bio.net Petah-Tikva, Israel; accessed on 21 July 2022); Alpha Bio SFB (www.alpha-bio.net Petah-Tikva, Israel; accessed on 21 July 2022); Alpha Bio SPI (www.alpha-bio.net Petah-Tikva, Israel; accessed on 21 July 2022); Argon K3pro Rapid (www.argon-dental.de Bingen am Rhein, Germany; accessed on 21 July 2022); Bego Semados RI (www.bego-implantology.com Bremen, Germany; accessed on 21 July 2022); Dentium Super Line (www.dentium.com Gyeonggi-do, South Korea; accessed on 21 July 2022); Friadent Ankylos C/X (www.dentsplysirona.com Warszawa, Poland; accessed on 21 July 2022); Implant Direct InterActive (www.implantdirect.com Thousand Oaks, United States of America; accessed on 21 July 2022); Implant Direct Legacy 3 (www.implantdirect.com Thousand Oaks, United States of America; accessed on 21 July 2022); MIS BioCom M4 (www.mis-implants.com Bar-Lev Industrial Park, Israel; accessed on 21 July 2022); MIS C1 (www.mis-implants.com Bar-Lev Industrial Park, Israel; accessed on 21 July 2022); MIS Seven (www.mis-implants.com Bar-Lev Industrial Park, Israel; accessed on 21 July 2022); MIS UNO One Piece (www.mis-implants.com Bar-Lev Industrial Park, Israel; accessed on 21 July 2022); Osstem Implant Company GS III (www.en.osstem.com Seoul, South Korea; accessed on 21 July 2022); SGS Dental P7N (www.sgs-dental.com Schaan, Liechtenstein; accessed on 21 July 2022); TBR Implanté (www.tbr.dental Toulouse, France; accessed on 21 July 2022); and Wolf Dental Conical Screw-Type (www.wolf-dental.com Osnabrück, Germany; accessed on 21 July 2022) (Table 4).

Surgery was performed under local anesthesia Artycaine + Adrenaline 1:100,000 by one surgeon (M.K) following the manufacturer-recommended protocols for dental implant placements. The healing process was carried out under a closed mucoperiosteal flap, unloaded in two-stage implants. Implants were loaded after 3 months of healing from the surgery. 

Standardized intraoral radiographs were taken immediately after surgery (00M) every 3 months in the first year of healing, every 6 months in the second year and every year until 5 years of observation (5y). Radiographs were taken using the DIGORA OPTIME radiography system (TYPE DXR-50, SOREDEX, Helsinki, Finland). The RTG images were taken in a standardized way [26] with the following parameters: 7 mA, 70 mV, and 0.1 s (the focus apparatus was from Instrumentarium Dental, Tuusula, Finland). Positioners were used to take images repeatably with a 90° angle of X-ray beam to the surface of phosphor plate.

Radiologically recorded peri-implant bone structure was studied by digital texture analysis using the Corticalization Index previously proposed [25,27] (as version 1 (CI)). It consists of the product of a measure that evaluates the number of long series of pixels of similar optical density with the mean optical density of the studied site (in the numerator) and the magnitude of the chaotic arrangement of the texture pattern, i.e., differential entropy (in the denominator).

**Figure 1:**
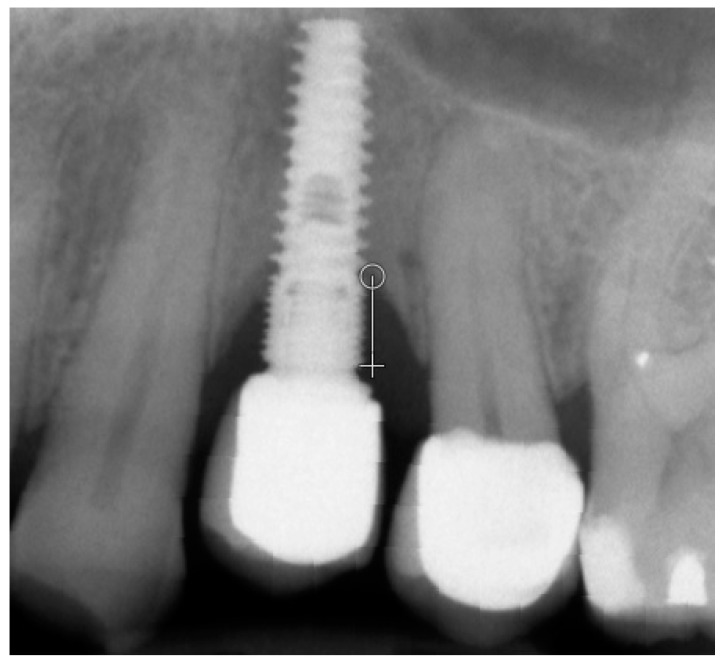
Measuring of marginal bone loss on the radiographic images 5 years after the functional loading. White line indicates the implant platform to the bottom of the bone loss cavity.

**Figure 2:**
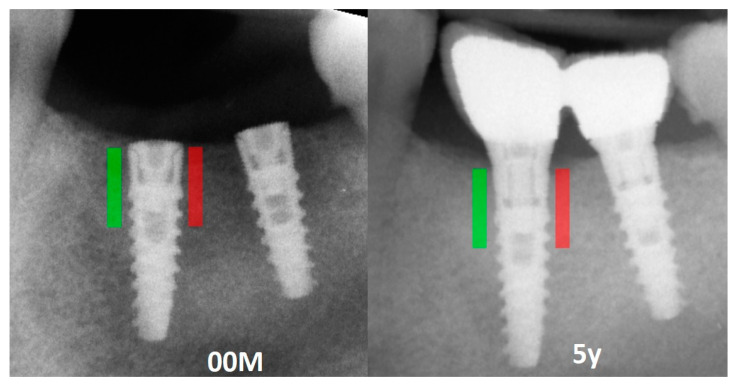
Marking an ROI. ROIs were marked near the implant neck area. Green area—mesial implant neck area; red area—distal implant neck area; Abbreviations: ROI—region of interest; 00M—0 months of observation; 5y—five years of observation.

Marginal bone loss (MBL) was measured on radiological images [28] (Figure 1) by only one researcher. Texture of X-ray images was analyzed in MaZda 4.6 freeware invented by University of Technology in Lodz [29,30] to test measures of corticalization in peri-implant environment of trabecular bone (representing original bone before implant-dependent alterations) and soft tissue (representing product of marginal bone loss). MaZda provides both first-order (mean optical density) and second-order (Differential Entropy: DifEntr, Long-Run Emphasis Moment: LngREmph) data. Due to the fact that the second-order data are given for four directions in the image, and in the present study, the authors do not wish to search for directional features, the arithmetic mean of these four primary data was included for further analysis. The regions of interest (ROIs) were marked near the neck area (Figure 2) and normalized (μ ± 3σ) to share the same mean (μ) and standard deviation (σ) of optical density within the ROI. To eliminate noise, [26] further worked on data reduced to 6 bits. For analysis in a co-occurrence matrix, a spacing of 5 pixels was chosen. In the formulas that follow, p(i) is a normalized histogram vector (i.e., histogram whose entries are divided by the total number of pixels in ROI), i = 1, 2,…, Ng, and Ng denotes the number of optical density levels. Mean optical density feature (only a first-order feature) was calculated as below:

$$\mathrm{Mean}\mathrm{Optical}\mathrm{Density}=\sum_{i=1}^{N_{g}}ip(i)$$

Second-order features:

$$DifEntr=-\sum_{i=1}^{Ng}p_{x-y}\left(i\right)log(p_{x-y}(i))$$

where Σ is the sum; Ng is the number of levels of optical density in the radiograph; i and j are the optical density of pixels with a 5-pixel distance from one another; p is probability; and log is common logarithm [31]. The differential entropy calculated in this way is a measure of the overall scatter of bone structure elements in a radiograph. Its high values are typical for cancellous bone [32,33,34,35]. Next, the last primary texture feature was calculated:

$$LngREmph=\frac{\sum_{i=1}^{Ng}\sum_{k=1}^{Nr}k^{2}p(i,k)}{\sum_{i=1}^{Ng}\sum_{k=1}^{Nr}p(i,k)}$$

where Σ is the sum; Nr is the number of series of pixels with density level i and length k; Ng is the number of levels for image optical density; Nr is the number of pixels in series; and p is probability [36,37]. This texture feature describes thick, uniformly dense, radio-opaque bone structures in intra-oral radiograph images [33,35].

$$CI=\frac{LngREmph\cdot \mathrm{Mean}\mathrm{Optical}\mathrm{Density}}{DifEntr}$$

Statistical analysis includes feature distribution evaluation, mean (*t*-test) or median (W-test) comparison, analysis of regression and one-way analysis of variance or Kruskal–Wallis test as non-normal distribution or between-group variance indicated on significant differences in investigated groups. Detected differences or relationships were assumed to be statistically significant when *p* < 0.05. Statgraphics Centurion version 18.1.12 (StatPoint Technologies, Warrenton, VA, USA) was used for statistical analyses.

## 3. Results

### 3.1. All Samples

**Figure 3:**
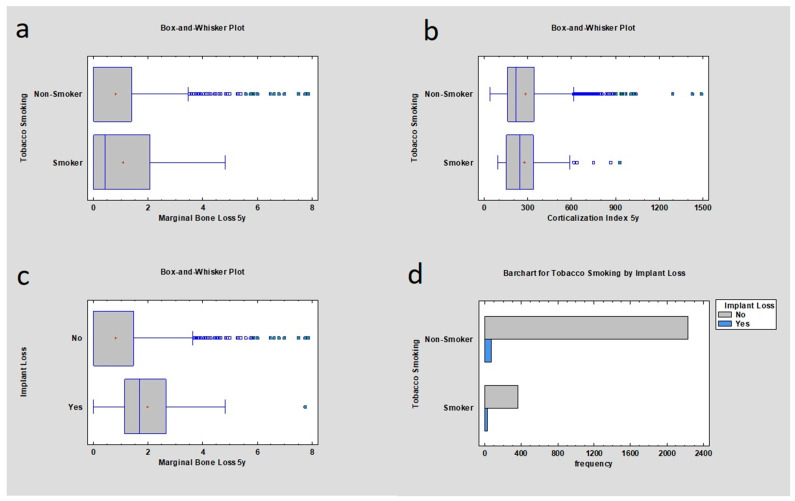
Dependences for all samples: (**a**) dependence of marginal bone loss from tobacco smoking after the dental implant insertion after 5 years of observation. The higher marginal bone loss was observed in the tobacco smoker group; (**b**) dependence of Corticalization Index from tobacco smoking after 5 years from implant insertion—there was no statistical difference; (**c**) dependence of implant loss from marginal bone loss after 5 years from functional loading—the higher marginal bone loss, the higher probability of implant loss; (**d**) frequency of implant loss depending on smoker or non-smoker group—the higher frequency was in the smoker group.

Statistical examination revealed that the initial MBL for the mandible and maxilla in the non-smoker group of samples was lower than in the group with smokers, respectively (mean 0 mm ± 0.85 mm; 0 mm ± 1.13 mm), which was statistically significant (*p* < 0.05). MBL after 5 years of observation was also correlated with nicotinismus according to statistics where *p* < 0.05, meaning there was statistical significance: MBL for non-smokers (mean 0 mm ± 1.25 mm) and MBL for smokers (mean 0.42 mm ± 1.32 mm). Corticalization Index for smokers and non-smokers was calculated immediately after the implant insertion and 5 years after the implantation; respectively, 185.98 ± 90.8 and 163.97 ± 151.9 for 00M; 243.17 ± 155.47 and 220.32 ± 184.97 after the 5 years. *p* value was lower than 0.05, which means it was statistically significant. Implant loss depending on MBL appearance after five years of observation was checked along with the value and analyzed for smokers and non-smoker group: 1.69 mm ± 1.73 mm and 0 mm ± 1.24 mm. *p* value was lower than 0.05. Implant loss frequency was checked. In a group of smokers, 6.74% of implants were lost; in a group of non-smoking patients, 2.87% of implants were lost, where the *p* value was lower than 0.05 (Table 5, Figure 3).

### 3.2. Mandible Group

**Figure 4:**
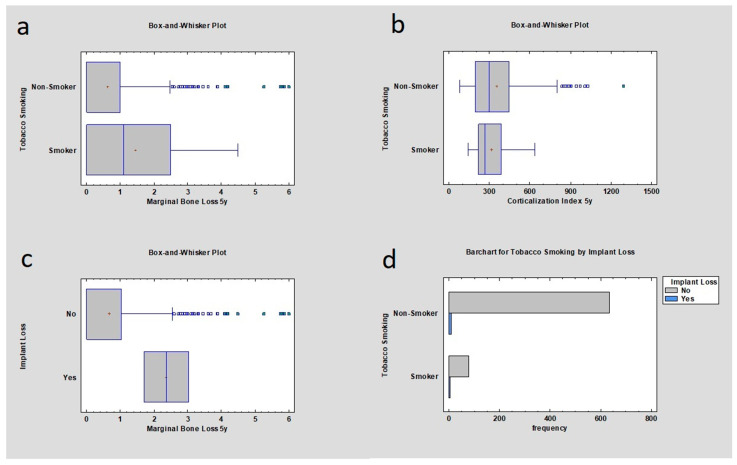
Dependencies for mandible samples: (**a**) dependence of marginal bone loss on tobacco smoking after the dental implant insertion after 5 years of observation. Higher marginal bone loss was observed in the tobacco smoker group; (**b**) dependence of the Corticalization Index on tobacco smoking 5 years after implant insertion—there was no statistical difference; (**c**) dependence of implant loss on marginal bone loss 5 years after functional loading—the higher the marginal bone loss, the higher probability of implant loss; (**d**) frequency of implant loss depending on the smoker or non-smoker group—the higher frequency was in the smoker group.

Research revealed that the initial MBL for implants in the mandible in the non-smoker group was lower than in the group with smokers, respectively (mean 0 mm ± 0.88 mm; 0 mm ± 1.83 mm), which was statistically significant (*p* < 0.05). MBL after 5 years of observation was also correlated with nicotinismus according to statistics where *p* < 0.05, which means that there was statistical significance: MBL for non-smokers (mean 0 mm ± 1.09 mm) and MBL for smokers (mean 1.10 mm ± 1.46 mm). Corticalization Index for smokers and non-smokers in the mandible implants group was calculated after the implant insertion and 5 years after the implantation, respectively, 195.81 ± 68.8 and 193.27 ± 136.54 for 00M; 263.87 ± 130.7 and 298.02 ± 200.1 after the 5 years. *p* value was higher than 0.05, which means it was not statistically significant. Implant loss depended on MBL appearance after five years of observation in a group of mandible-inserted implants; the value was checked and analyzed for smokers and non-smoker group: 2.36 mm ± 0.94 mm and 0 mm ± 1.13 mm. *p* value was lower than 0.05. The relation between implant loss and the occurrence of corticalization was analyzed. Respectively, for smokers and non-smokers, values for initial corticalization were 183.62 ± 18 and 194.10 ± 131.65; 5 years after the implantation, values were 438.18 ± 341.70 for smokers and 292.40 ± 193.95 for non-smokers, and the *p* value was higher than 0.05. Implant loss frequency was also checked for mandible implants in relation to smoking. In a group of smokers, 1.66% of implants were lost; in a group of non-smoking patients, 1.25% of implants were lost; the relationship was weak, but the *p* value was still lower than 0.05 (Table 6, Figure 4).

### 3.3. Maxilla Group

**Figure 5:**
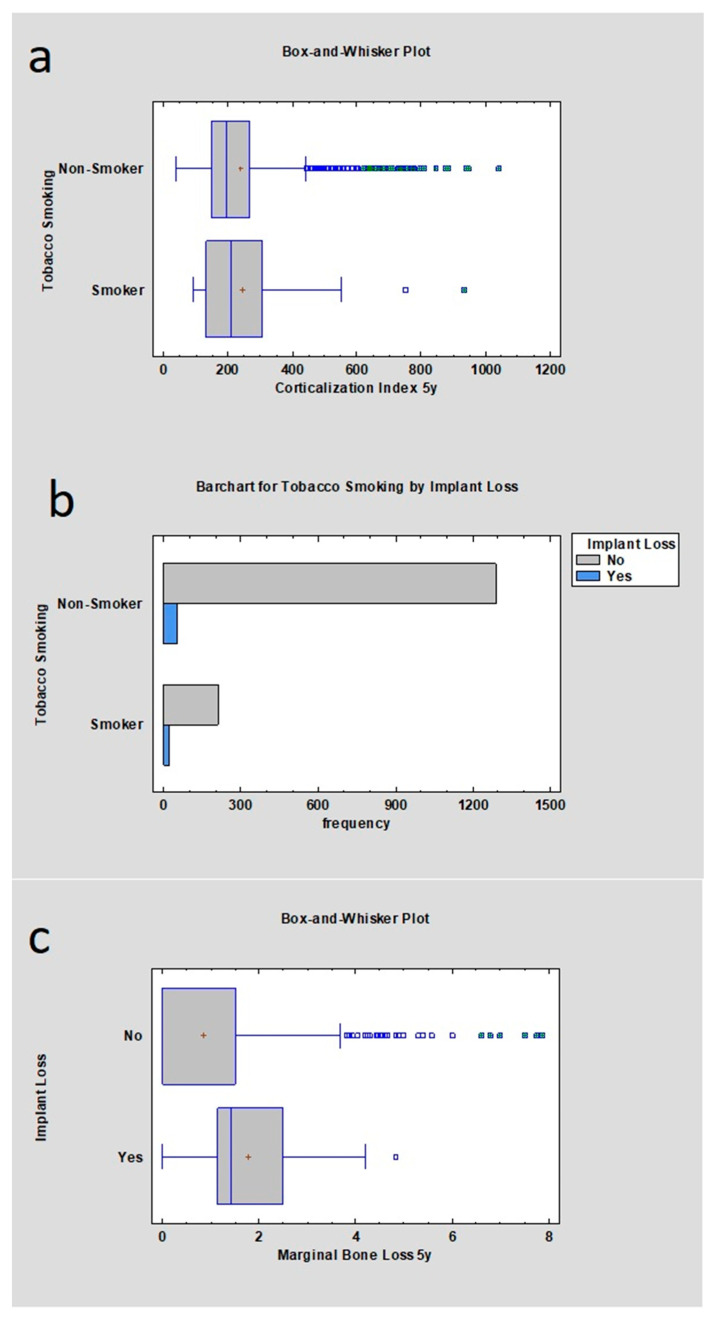
Dependencies for maxilla samples: (**a**) dependence of the Corticalization Index on tobacco smoking 5 years after implant insertion—there was no statistical difference; (**b**) frequency of implant loss depending on the smoker or non-smoker group—the higher frequency was in the smoker group; (**c**) dependence of implant loss on marginal bone loss a 5 years after functional loading—the higher the marginal bone loss, the higher the probability of implant loss.

Implants in the maxilla were also examined separately. Statistics showed that the initial MBL for implants in the maxilla in the non-smoker group was lower than in the group with smokers, respectively (mean 0 mm ± 0.82 mm; 0 mm ± 2.38 mm), which was statistically significant (*p* < 0.05). MBL after 5 years of observation was also correlated with nicotinismus according to statistics where *p* < 0.05, meaning there was statistical significance: MBL for non-smokers (mean 0 mm ± 1.29 mm) and MBL for smokers (mean 1.42 mm ± 1.34 mm). The Corticalization Index for smokers and non-smokers in the maxilla implants group was calculated after implant insertion and 5 years after implantation; respectively, 173.50 ± 92.80 and 146.56 ± 109.65 for 00M, where the *p* value was lower than 0.05; 207.06 ± 153.50 and 193.68 ± 151.10 after 5 years. The *p* value was higher than 0.05, meaning there was no statistical significance. Implant loss, depending on MBL appearance after five years of observation in the group of maxilla inserted implants, was checked and analyzed for smokers and non-smoker groups: 1.42 mm ± 1.34 mm and 0 mm ± 1.29 mm. *p* value was lower than 0.05. In this group, the relation between implant loss and the occurrence of corticalization was also analyzed. Respectively, for smokers and non-smokers, values for initial corticalization were 173.56 ± 70.82 and 147.20 ± 109.40; and 5 years after implantation, 235.15 ± 268.71 for smokers and 193.67 ± 147.98 for non-smokers. The *p* value was higher than 0.05. Implant loss frequency was also checked for maxilla implants in relation to smoking. In the group of smokers, 4.34% of implants were lost; conversely, in the group of non-smoking patients, 1.49% of implants were lost. The relationship between smoking and implant loss was weak, but the *p* value was still lower than 0.05 (Table 7, Figure 5).

### 3.4. Corticalization vs. Torque

**Figure 6:**
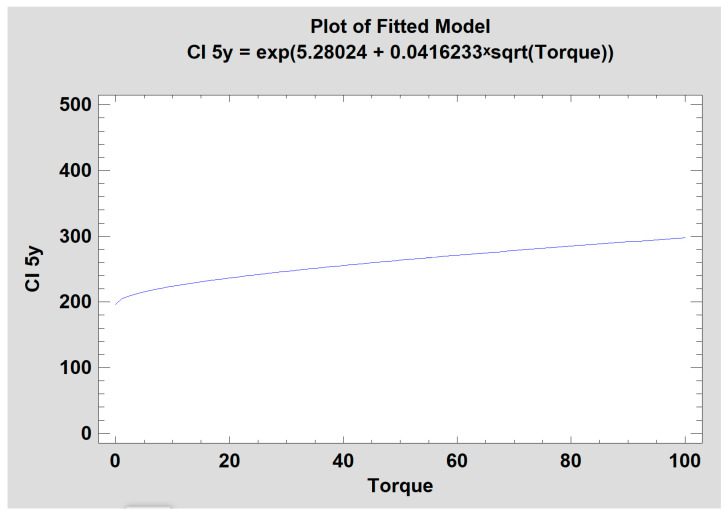
Dependence of the Corticalization Index on torque value after dental implant insertion, following 5 years of observation. The higher the torque during dental implantation, the higher the Corticalization Index after 5 years.

The study also revealed a correlation between the Corticalization Index and the torque used during implant insertion (when the torque value was increasing), and the probability of occurrence of the corticalization phenomenon was also higher. The *p* value was lower than 0.05 (Figure 6).

## 4. Discussion

The question that arises is the following: Is corticalization in radiographs associated with a higher risk of bone loss around dental implants in smoking patients? Until now, there have been no publications that can confirm or deny this. In this research, 768 patients with dental implants were studied, and 2196 samples of the neck implant area were analyzed. The study showed that the Corticalization Index is real and changes throughout the healing period, and the value of this index varies depending on the groups analyzed (smoking and non-smoking patients). The presented research shows that there is a close correlation between smoking and changes in bone structure. This correlation was calculated and discovered at the pixel level, taking into account texture features. Marginal bone loss has been analyzed many times, but no one has ever checked the bone structure and its relations to nicotinism at the pixel level [38,39,40].

Taking into account the corticalization phenomenon in all analyzed implants, a higher index was observed in the group of smokers already at the time of implantation (Table 5). A higher index of corticalization was observed in the group of smokers on the day of surgery, and a smaller index was observed in the group where patients did not smoke (bone changes at the pixel level were observed before implant placement, this may confirm that the tobacco smoking habit leads to undesirable changes in bone morphology). The Corticalization Index increases throughout the healing process. It was also observed in the mandible and maxilla separately, but in the case of the maxilla, the difference was greater. Corticalization is a process that changes the structure of the bone, where the trabecular bone is replaced by cortical bone. Remodeling depends on resorption and new bone formation [41,42,43]. Cancellous bone is composed of trabeculae with marrow between them. Corticalization is the process that changes trabecular bone into denser tissue—cortical [44].

Marginal bone loss is also correlated with nicotinismus, a fact that is commonly known and confirmed in this research. This study affirmed that marginal bone loss around dental implants is associated with tobacco smoking, as analyzed through radiographs and texture features in a large group of implants. Further research showed that MBL is higher in implants that were inserted in the maxilla in the smoker group, as opposed to implants inserted in the mandible. It can be concluded that smoking has an influence on bone structure before implantation and leads to a higher value of the Corticalization Index. The effect of this correlation may result in marginal bone loss. After five years of observation, the research showed that the higher the MBL, the greater the probability of implant loss in the smoking patients’ group, which is indirectly correlated with the occurrence of the corticalization phenomenon [24,25,27].

Directly in statistics, there is no statistical significance between smoking and corticalization, but a similarity can be suspected. The Corticalization Index is consistent after 5 years. Smoking is a factor that can change the structure of bone tissue at the cellular level. Changes depend on disturbances in angiogenesis, impaired vascularization and nutrition of bone cells, with an impact on bone metabolism and osteogenesis. If the tissue is not nourished, it atrophies [45,46,47,48]. The study presented shows a close relationship between smoking and MBL and also between smoking and implant failure after 5 years of observation. The research emphasizes the impact of smoking on dental implant loss, mainly in the maxilla. Implant loss frequency was almost two times higher than in the case of mandible implants. All these correlations were detected at the pixel level using only texture features and by calculating and analyzing the Corticalization Index.

Additionally, the appearance of corticalization after 5 years of observation was also correlated with the increasing torque value during the implantation procedure. Recent studies have proved that a high value of torque during implant insertion leads to MBL [49,50,51]. There is a probability that condensed bone around the dental implant, caused by a high torque value, is not a desirable effect. Currently, the popular preparation method and implantation technique is osseodensification [52,53,54]. Osseodensification around dental implants increases the primary stability of the implant, but if corticalization may lead to peri-implantitis, marginal bone loss, and consequently dental implant loss, what will be the long-term consequences of densifying bone tissue next to the implant body? This phenomenon should be a focus of future research.

Taking into account the types of implants, it can be stated that not only the design of the dental implant affects the corticalization phenomenon. There is a relationship between bone condition, prosthetic restoration and also the surgeon’s experience and preferences [24,55,56].

The corticalization phenomenon, as analyzed in this research, is closely related to smoking, marginal bone loss and implant loss. This phenomenon may be indicated before implant placement and also before marginal bone loss during the control periods when RTG images are taken. Considering the clinical relevance of this research, if there are enough studies about this phenomenon and it becomes well-known, it may become a useful tool for prognosis, contraindications and marginal bone loss prevention. Until now, there have been many tools, as mentioned above, but never at the pixel level [57,58,59].

Two-dimensional RTG images were taken in a defined period of observation. If the general condition of the patient changes, it may have an impact on bone structure. This can also be observed in the textures of RTG images. A limitation of this study was that laboratory tests were not checked after the implant insertion throughout the observation period. This should be taken into consideration in future research [60].

## 5. Conclusions

The higher Corticalization Index that occurs is related to tobacco smoking, even as early as the day of surgery. This is the latest study that confirms the close and non-beneficial impact of the corticalization phenomenon on the implant region, leading to peri-implantitis. This research demonstrated and proved that smoking tobacco has an impact on bone structure, which can be identified at the pixel level without clinical examination, using only two-dimensional radiographs. 

The corticalization phenomenon may not be directly related to implant loss after 5 years of observation, but a relationship is observed between corticalization and marginal bone loss after 5 years, which may lead to peri-implantitis and implant loss. 

The uniqueness of this research is that all these dependencies can be diagnosed by analyzing texture features in RTG images.

## Figures and Tables

**Table 1:** Inclusion criteria for the research.

Inclusion Criteria
18 years of age
Bleeding upon gingival probing < 20%
Probing depth ≤ 3 mm
Good oral hygiene
Regular follow-ups
Two-dimensional radiographs taken during the regular check
Laboratory test: • PTH, where the norm is from 10 to 60 pg/mL); • TSH, where the norm is 0.23–4.0 µU/mL; • Calcium in serum (Ca^2+^), where the norm is 9–11 mg/dL; • HbA1c, where the norm is <5%; • Vitamin 25(OH)D3 (D3), where the norm is 31–50 ng/mL.
Spine densitometry
Smoking 1 or more cigarettes per day

**Table 2:** Exclusion criteria for the research.

Exclusion Criteria
Lack of X-ray images
Defective X-ray images in the visual assessment
Lack of laboratory tests
Uncontrolled internal co-morbidity: • Diabetes mellitus; • Thyroid dishormonoses; • Rheumatoid disease; • Other immunodeficiencies.
History of oral radiation therapy
Past or current use of cytostatic drugs
Soft and bone tissue augmentation
Low-quality or lack of follow-up radiographs

**Table 3:** Limitation of the research.

Limitations of the Study
Laboratory tests have not been carried out 3 months after the research.

**Table 4:** The implant types used and their features.

Implant Name	Titanium Alloy No.	Insertion Level	Connection Type	Connection Shape	Neck Shape	Neck Microthread	Body Shape	Body Thread	Apex Shape	Apex Hole	Apex Groove
AB Dental Devices I5	Grade 5	Bone level	Internal	Hexagon	Straight	No	Tapered	Square	Flat	No hole	Yes
ADIN Dental Implants Touareg	Grade 5	Bone level	Internal	Hexagon	Straight	Yes	Tapered	Square	Flat	No hole	Yes
Alpha Bio ATI	Grade 5	Bone level	Internal	Hexagon	Straight	Yes	Straight	Square	Flat	No hole	Yes
Alpha Bio OCI	Grade 5	Bone level	Internal	Hexagon	Straight	No	Straight	No Threads	Dome	Round	No
Alpha Bio DFI	Grade 5	Bone level	Internal	Hexagon	Straight	Yes	Tapered	Square	Flat	No hole	Yes
Alpha Bio SFB	Grade 5	Bone level	Internal	Hexagon	Straight	No	Tapered	V-shaped	Flat	No hole	Yes
Alpha Bio SPI	Grade 5	Bone level	Internal	Hexagon	Straight	Yes	Tapered	Square	Flat	No hole	Yes
Argon Medical Prod. K3pro Rapid	Grade 4	Subcrestal	Internal	Conical	Straight	Yes	Tapered	V-shaped	Dome	No hole	Yes
Bego Semados RI	Grade 4	Bone level	Internal	Hexagon	Straight	Yes	Tapered	Reverse buttress	Cone	No hole	Yes
Dentium Super Line	Grade 5	Bone level	Internal	Conical	Straight	No	Tapered	Buttress	Dome	No hole	Yes
Friadent Ankylos C/X	Grade 4	Subcrestal	Internal	Conical	Straight	No	Tapered	V-shaped	Dome	No hole	Yes
Implant Direct InterActive	Grade 5	Bone level	Internal	Conical	Straight	Yes	Tapered	Reverse buttress	Dome	No hole	Yes
Implant Direct Legacy 3	Grade 5	Bone level	Internal	Hexagon	Straight	Yes	Tapered	Reverse buttress	Dome	No hole	Yes
MIS BioCom M4	Grade 5	Bone level	Internal	Hexagon	Straight	No	Straight	V-shaped	Flat	No hole	Yes
MIS C1	Grade 5	Bone level	Internal	Conical	Straight	Yes	Tapered	Reverse buttress	Dome	No hole	Yes
MIS Seven	Grade 5	Bone level	Internal	Hexagon	Straight	Yes	Tapered	Reverse buttress	Dome	No hole	Yes
Osstem Implant Company GS III	Grade 5	Bone level	Internal	Conical	Straight	Yes	Tapered	V-shaped	Dome	No hole	Yes
SGS Dental P7N	Grade 5	Bone level	Internal	Hexagon	Straight	Yes	Tapered	V-shaped	Flat	No hole	Yes
TBR Implanté	Grade 5	Bone level	Internal	Octagon	Straight	No	Straight	No threads	Flat	Round	Yes
Wolf Dental Conical Screw-Type	Grade 4	Bone level	Internal	Hexagon	Straight	No	Tapered	V-shaped	Cone	No Hole	Yes

**Table 5:** Table presents values for marginal bone loss, values of Corticalization Index and implant loss frequency. Values were calculated for all the implantations for smokers and non-smokers. 00M—the observation period immediately after the implantation; 5y—the observation period 5 years after the implantation.

	Observation Period	Smoker	Non-Smoker	*p* Value
Smoking/Marginal Bone Loss	00M	0 mm ± 1.13 mm	0 mm ± 0.85 mm	*p* < 0.05
	5y	0.42 mm ± 1.32 mm	0 mm ± 1.25 mm	*p* < 0.05
Smoking/Corticalization Index	00M	185.98 ± 90.8	163.97 ± 151.9	*p* < 0.05
	5y	243.17 ± 155.47	220.32 ± 184.97	*p* = 0.85
Implant Loss to Marginal Bone Loss	5y	1.69 mm ± 1.73 mm	0 mm ± 1.24 mm	*p* < 0.05
Implant Loss Frequency	5y	6.74%	2.87%	*p* < 0.05

**Table 6:** Table presents values for marginal bone loss, values of Corticalization Index and implant loss frequency. Values were calculated for implants in mandible for smokers and non-smokers. 00M—the observation period immediately after the implantation; 5y—the observation period 5 years after the implantation.

	Observation Period	Smoker	Non-Smoker	*p* Value
Smoking/Marginal Bone Loss	00M	0 mm ± 1.83 mm	0 mm ± 0.88 mm	*p* < 0.05
	5y	1.10 mm ± 1.46 mm	0 mm ± 1.09 mm	*p* < 0.05
Smoking/Corticalization Index	00M	195.81 ± 68.8	193.27 ± 136.54	*p* = 0.85
	5y	263.87 ± 130.7	298.02 ± 200.1	*p* = 0.68
Implant Loss to Marginal Bone Loss	5y	2.36 mm ± 0.94 mm	0 mm ± 1.13 mm	*p* < 0.05
Implant Loss Frequency	5y	1.66%	1.25%	*p* < 0.05

**Table 7:** This table presents values for marginal bone loss, Corticalization Index and implant loss frequency. Values were calculated for implants in maxilla for smokers and non-smokers. 00M—the observation period immediately after implantation; 5y—the observation period 5 years after implantation.

	Observation Period	Smoker	Non-Smoker	*p* Value
Smoking/Marginal Bone Loss	00M	0 mm ± 2.38 mm	0 mm ± 0.82 mm	*p* < 0.05
	5y	1.42 mm ± 1.34 mm	0 mm ± 1.29 mm	*p* < 0.05
Smoking/Corticalization Index	00M	173.50 ± 92.80	146.56 ± 109.65	*p* < 0.05
	5y	207.06 ± 153.50	193.68 ± 151.10	*p* = 0.81
Implant Loss to Marginal Bone Loss	5y	1.42 mm ± 1.34 mm	0 mm ± 1.29 mm	*p* < 0.05
Implant Loss Frequency	5y	4.34%	1.49%	*p* < 0.05

## Data Availability

The data on which this study is based will be made available upon request at https://www.researchgate.net/profile/Tomasz-Wach.
